# Roles of ST2, IL‐33 and BNP in predicting major adverse cardiovascular events in acute myocardial infarction after percutaneous coronary intervention

**DOI:** 10.1111/jcmm.13183

**Published:** 2017-06-17

**Authors:** Yan‐Peng Wang, Jian‐Hua Wang, Xiao‐Long Wang, Jun‐Yi Liu, Fang‐Yun Jiang, Xiao‐Li Huang, Jing‐Yu Hang, Wei Qin, Shi‐Xin Ma, Jie Zhang, Min‐Jie Yuan, Jing‐Bo Li, Zhi‐Gang Lu, Meng Wei

**Affiliations:** ^1^ Department of Cardiology Shanghai Jiao Tong University Affiliated Sixth People's Hospital Shanghai China; ^2^ Clinical Laboratory Fu Yang People's Hospital Anhui China; ^3^ Department of Cardiology Hong Si Bao People's Hospital Wuzhong Ningxia Hui Autonomous Region China; ^4^ Shanghai Runda Rongjia Biological Company Ltd. Shanghai China

**Keywords:** ST2, IL‐33, BNP, Major adverse cardiovascular events, Acute myocardial infarction, Percutaneous coronary intervention

## Abstract

This study investigated roles of serum ST2, IL‐33 and BNP in predicting major adverse cardiovascular events (MACEs) in acute myocardial infarction (AMI) after percutaneous coronary intervention (PCI). Blood samples were collected from the included AMI patients (*n* = 180) who underwent PCI. All patients were divided into the MACEs and MACEs‐free groups. Enzyme‐linked immunosorbent assay was performed to measure serum levels of ST2, IL‐33 and BNP. Severity of coronary artery lesion was evaluated by Gensini score. Pearson correlation analysis was used. A receiver operating characteristics curve was drawn to evaluate the potential roles of ST2, IL‐33 and BNP in predicting MACEs, and Kaplan–Meier curve to analyse the 1‐year overall survival rate. Logistic regression analysis was conducted to analyse the independent risk factors for MACEs. Compared with the MACEs‐free group, the serum levels of ST2, IL‐33 and BNP were significantly higher in the MACEs group. Serum levels of ST2, IL‐33 and BNP were positively correlated with each other and positively correlated with Gensini score. The area under curves of ST2, IL‐33 and BNP, respectively, were 0.872, 0.675 and 0.902. The relative sensitivity and specificity were, respectively, 76.27% and 85.92%, 69.49% and 58.68%, as well as, 96.61% and 77.69%. Serum levels of ST2, IL‐33 and BNP were independent risk factors for MACEs. The 1‐year overall survival rate was higher in AMI patients with lower serum levels of ST2, IL‐33 and BNP. In conclusion, serum levels of ST2, IL‐33 and BNP have potential value in predicting MACEs in AMI patients undergoing PCI.

## Introduction

AMI is the most common and dangerous cardiac emergency with high mortality worldwide, which is commonly known as a heart attack occurring when blood stops flowing to a portion of the heart, causing damage to the heart muscle due to not receiving enough oxygen 1, 2, 3. With the speedup of ageing population, the incidence of AMI has been increasing in recent years and has been an important cause of heart failure 3, 4, 5. Only thrombolytic therapy and primary or rescue PCI are known to be effective in the management of AMI 6. PCI is reported to be safe and can help to restore blood flow 7. However, AMI has a not very good long‐term prognosis and an enormous amount of patients suffer from MACEs, such as all‐cause death, stent thrombosis, myocardial infarction and target vessel revascularization 8, 9. Therefore, developing novel diagnostic and prognostic biomarkers for AMI is essential.

Serum ST2, the most prominent orphan member of interleukin‐1 (IL‐1) receptor, has been recognized to negatively regulate Toll‐like receptor/IL‐1 receptor signalling and function as a critical effector molecule of T helper type 2 (TH2) responses 10, 11. ST2 gene products mainly include two distinct isoforms, transmembrane form (ST2L) and soluble secreted form (sST2) 12. ST2 has been reported to be a significantly prognostic biomarker for MACEs of AMI 13. And sST2 shows the function of a decoy receptor, which neutralizes the benefits of IL‐33 and thus play a deleterious role, and additionally, it can reduce the hypertrophic and/or fibrotic cardiac responses to mechanical stress together with interleukin‐33 (IL‐33) 14. IL‐33, which is thought to be the functional ligand of sST2 recently, is protective of the heart, and the IL‐33 ratio also correlates with the prognosis of AMI patients 1, 10. B‐type natriuretic peptide (BNP), as a novel cardiac hormone in humans, is synthesized in ventricular myocardium, and most of BNP secreted into the circulation emerges through the coronary sinus from the heart 15. In addition, BNP is widely recognized as a marker for multiple cardiovascular diseases and has been reported to have negative correlations with the prognosis of AMI 16, 17. Increasing evidence has indicated that serum levels of ST2, IL‐33 and BNP have independent values in predicting the prognosis of AMI patients 18, 19, 20. But direct relations between the serum levels of ST2, IL‐33 and BNP and the occurrence MACEs in AMI patients have been rarely reported. Thus, the present study tried to explore the predictive values of serum ST2, IL‐33 and BNP levels for MACEs in AMI patients undergoing PCI.

## Materials and methods

### Study participants

From January 2014 to June 2015, 180 patients (110 males and 70 females; mean age 61.41 ± 8.90 years) diagnosed as a first episode of AMI in the Department of Cardiology in Shanghai Jiao Tong University Affiliated Sixth People's Hospital were enrolled in this study. The patients underwent PCI successfully. Clinical data of all patients were recorded. All patients were in accordance with the diagnostic criteria for AMI by American College of Cardiology (ACC) or American Heart Association (AHA) 21: (*i*) chest pain lasting more than 30 min.; (*ii*) the dynamic electrocardiogram change; (*iii*) levels of serum markers of myocardial injury were changed; (*iv*) the coronary angiography showed that patients could receive PCI for anatomy in infarct‐related artery. Exclusion criteria: (*i*) patients with survival time less than 24 hrs after onset; (*ii*) patients with other heart disease, combined acute and chronic infection; (*iii*) patients with abnormal pulmonary artery and/or aorta; (*iv*) patients with liver and kidney dysfunctions or malignant tumours; (*v*) patients who underwent cardiology‐related surgery before; (*vi*) patients with serum creatinine more than 3.0 mg/dl; and (*vii*) patients whose recent treatment may affect serum levels of ST2, IL‐33 and BNP. All patients were divided into the MACEs group (AMI patients with MACEs) and the MACEs‐free group (AMI patients without MACEs). MACEs include nonfatal myocardial infarction, angina, malignant arrhythmia and death 22. The study was approved by the Ethics Committee of Shanghai Jiao Tong University Affiliated Sixth People's Hospital, and all the study subjects have prior to the study signed the written informed consent.

### PCI

All AMI patients received PCI surgery as soon as possible after admission to open the infarct‐related artery. The patients were given 300 mg of aspirin and Plavix orally. Routine coronary angiography was conducted to determine the infarction and stenosis. The conventional PCI surgery was performed, and the vascular patency rate of all patients was 100%. Blood flow reached thrombolysis in myocardial infarction (TIMI) grade III. The colour Doppler echocardiography was conducted 1 week after surgery. After discharge, all patients were treated in accordance with the standard treatment of myocardial infarction 23.

### Coronary angiography

Patients received coronary angiography immediately after admission, which was performed in the cath laboratory, with a digital subtraction machine (Artiszee AXIOM; Siemens Ltd, Erlangen, Germany). After conventional disinfection and local anaesthesia, the patients were supine; the catheter was from the puncture artery to the left and right coronary artery. Coronary angiography was conducted from multiple angles, including more than three positions above the right coronary artery, and more than six positions above left coronary artery. Other positions were selected if necessary. The coronary angiography was performed by cardiologists in Shanghai Jiao Tong University Affiliated Sixth People's Hospital. Meanwhile, two interventional cardiologists judged the stenosis of coronary artery by double‐blind method, which was calculated by the percentage of 1 (internal diameter of the stenosis/internal diameter of the normal coronary artery adjacent to the stenosis in the proximal and distal part).

### Severity of coronary artery lesion evaluated by Gensini score

Different weight coefficients were given according to the location of the diseased vessels. Weight coefficient for left main vessel is 5; with 2.5 for proximal lesion of the left circumflex and the anterior descending branch of circumflex, 1.5 for middle segments of anterior descending lesions, 1 for the right coronary artery, anterior descending artery, diagonal branch, posterior branch of left ventricle and obtuse marginal branch, and 0.5 for other vessels. According to the degree of vascular stenosis, 1 point indicates <25%, 2 points for 26–50%, 4 points for 51–75%, 8 points for 76–90%, 16 points for 91–99% and 32 points for 100%. The total score of each branch blood vessel = scores of the stenosis degree × weight coefficient 24.

### Baseline characteristics and blood sample collection

The age, gender, other baseline characteristics, heart rate, history of hypertension, diabetes mellitus and smoking of all AMI patients were recorded. Two tubes of venous blood (each 4 ml) of all AMI patients in the fasting state were collected, one of which was used for routine blood examination, to measure blood urea nitrogen (BUN), creatinine (Cr), uric acid (UA), high‐sensitive C‐reactive protein (hs‐CRP), total cholesterol (TC), low‐density lipoprotein cholesterol (LDL‐C), triglyceride (TG), high‐density lipoprotein cholesterol (HDL‐C) and other parameters. The other tube of blood was centrifuged under the condition of 3000 *r*/min for 10 min. at 4°C. The serum was separated and then stored at −80°C for the detection of ST2, IL‐33 and BNP levels.

### Enzyme‐linked immunosorbent assay (ELISA)

The serum levels of ST2 and IL‐33 were detected using ELISA Kit (batch number: mL078255 and mL03902; Abbott Laboratories, Abbott Park, IL, USA). The serum level of BNP was detected using human brain natriuretic peptide ELISA Kit (mk25022; Getein Biotech, Inc., Nanjing, China) in strict accordance with the kit instructions. The ELISA Kit was placed at room temperature for 30 min. The standard product was diluted according to the instructions, prepared for use. The serum was thawed at room temperature for 30 min., followed by adding samples, mixing liquid, washing, coloration and the termination. The microplate reader (SAF‐680T; BAJU Co. Ltd., Shanghai, China) was finally used to determine optical density (OD) value under 450 nm. Linear regression equation for the standard curve was calculated according to the standard OD values. Next, the actual concentrations were calculated.

### Follow‐up

All the patients were followed up for 1 year by outpatient or telephone, with the follow‐up rate 100%. The occurrence of MACEs and the survival of all patients were recorded.

### Statistical analysis

SPSS 20.0 statistical software (SPSS Inc., Chicago, IL, USA) was used for statistical analysis. Measurement data were expressed as mean ± standard deviation (mean ± S.D.). Comparison between two groups was made using independent sample *t*‐test. Enumeration data were presented as percentage or rate, which was analysed by chi‐square test. Pearson correlation analysis was performed to analyse the correlations among serum ST2, IL‐33 and BNP levels and the correlations of serum ST2, IL‐33 and BNP levels with Gensini scores. Receiver operating characteristics (ROC) curve was drawn to evaluate the potential roles of ST2, IL‐33 and BNP in predicting MACEs. Logistic regression analysis was conducted to analyse the independent risk factors for MACEs in AMI patients. Kaplan–Meier curve was adopted to analyse the 1‐year overall survival rate of AMI patients. *P *<* *0.05 was considered statistically significant.

## Results

### Baseline characteristics of AMI patients in the MACEs and MACEs‐free groups

Statistical analysis revealed that the occurrence of post‐operative MACEs had no correlations with age, gender, body mass index (BMI), hypertension history, diabetes mellitus history, smoking history, heart rate, systolic blood pressure (SBP), diastolic blood pressure (DBP), TC, TG, LDC‐C, HDL‐C, troponin I (cTn I) and creatine kinase‐MB (CK‐MB) (all *P *>* *0.05), while it had correlations with the Gensini score at admission and hs‐CRP (both *P *<* *0.05; Table 1).

**Table 1:** Baseline characteristics of AMI patients in the MACEs and MACEs‐free groups

Characteristic	MACEs group (*n* = 59)	MACEs‐free group (*n* = 121)	*P*
Gender (male/female)	38/21	72/49	0.527
Age (years)	61.93 ± 9.29	61.15 ± 8.74	0.583
BMI (kg/m^2^)	24.47 ± 2.63	25.12 ± 2.59	0.118
Hypertension history (%)	38 (64.41)	60 (49.59)	0.061
Diabetes mellitus history (%)	25 (42.37)	56 (46.28)	0.621
Smoking history (%)	36 (61.02)	58 (47.93)	0.099
Gensini score	82.39 ± 28.92	69.76 ± 21.28	0.001
Heart Rate (beats/min)	75.49 ± 5.27	74.28 ± 4.87	0.130
SBP (mmHg)	129.53 ± 13.47	132.64 ± 13.75	0.153
DBP (mmHg)	74.68 ± 7.25	75.22 ± 8.11	0.665
TC (mmol/l)	4.59 ± 0.47	4.69 ± 0.49	0.195
TG (mmol/l)	1.74 ± 0.18	1.78 ± 0.19	0.179
LDC‐C (mmol/l)	3.23 ± 0.32	3.31 ± 0.34	0.133
HDL‐C (mmol/l)	1.15 ± 0.13	1.12 ± 0.11	0.108
hs‐CRP (mg/l)	7.38 ± 1.13	7.03 ± 1.03	0.039
cTn I (ng/ml)	4.62 ± 1.49	4.73 ± 1.45	0.636
CK‐MB (mmol/l)	178.55 ± 50.63	197.24 ± 68.98	0.065

AMI, acute myocardial infarction; MACEs, major adverse cardiovascular events; BMI, body mass index; SBP, systolic blood pressure; DBP, diastolic blood pressure; TC, total cholesterol; TG, triglyceride; LDL‐C, low‐density lipoprotein cholesterol; HDL‐C, high‐density lipoprotein cholesterol; hs‐CRP, high‐sensitivity C‐reactive protein; cTn I, cardiac troponin I; CK‐MB, creatine kinase‐MB.

### Comparisons of serum levels of ST2, IL‐33 and BNP in AMI patients at admission between the MACEs and MACEs‐free groups

**Figure 1:**
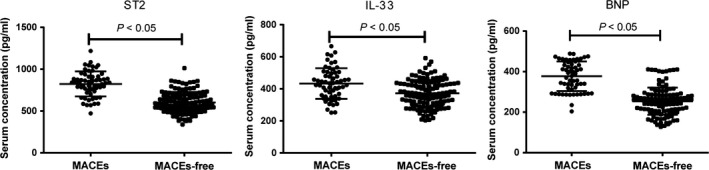
Comparisons of serum levels of ST2, IL‐33 and BNP in AMI patients at admission between the MACEs and MACEs‐free groups. AMI, acute myocardial infarction; MACEs, major adverse cardiovascular events; ST2, homolog of sulfotransferase; IL‐33, interleukin‐33; BNP, B‐brain natriuretic peptide; *, compared with the MACEs‐free group, *P *<* *0.05.

The serum levels of ST2, IL‐33 and BNP in the MACEs group at admission were 823.47 ± 149.25 pg/ml, 433.27 ± 95.64 pg/ml and 377.21 ± 73.2 pg/ml, respectively. The serum levels of ST2, IL‐33 and BNP in the MACEs‐free group before undergoing PCI were 601.83 ± 125.84 pg/ml, 372.25 ± 84.25 pg/ml and 253.99 ± 66.68 pg/ml, respectively. The serum levels of ST2, IL‐33 and BNP were significantly different between the two groups (all *P *<* *0.05; Fig. 1).

### Correlations of serum levels of ST2, IL‐33 and BNP in AMI patients

**Figure 2:**
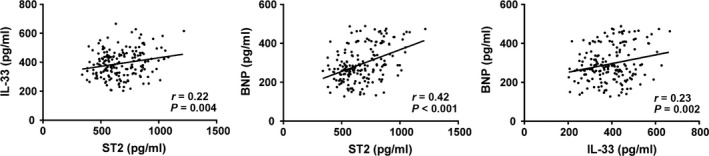
Correlations of serum levels of ST2, IL‐33 and BNP in AMI patients. AMI, acute myocardial infarction; ST2, homolog of sulfotransferase; IL‐33, interleukin‐33; BNP, B‐brain natriuretic peptide.

Pearson correlation analysis showed that serum levels of ST2, IL‐33 and BNP were positively correlated with each other in all AMI patients (*r* = 0.22, *r* = 0.42, *r* = 0.23, all *P *<* *0.05; Fig. 2).

### Correlations of serum levels of ST2, IL‐33 and BNP with Gensini score in AMI patients

**Figure 3:**
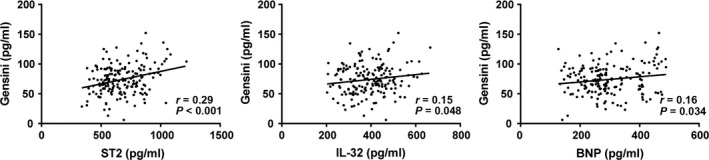
Correlations of serum levels of ST2, IL‐33 and BNP with Gensini score in AMI patients. AMI, acute myocardial infarction; ST2, homolog of sulfotransferase; IL‐33, interleukin‐33; BNP, B‐brain natriuretic peptide.

Pearson correlation analysis revealed that the serum levels of ST2, IL‐33 and BNP had positive correlations with Gensini score (*r* = 0.29, *r* = 0.15, *r* = 0.16, all *P *<* *0.05; Fig. 3).

### ROC curve analysis on predictive values of serum levels of ST2, IL‐33 and BNP for MACEs

**Figure 4:**
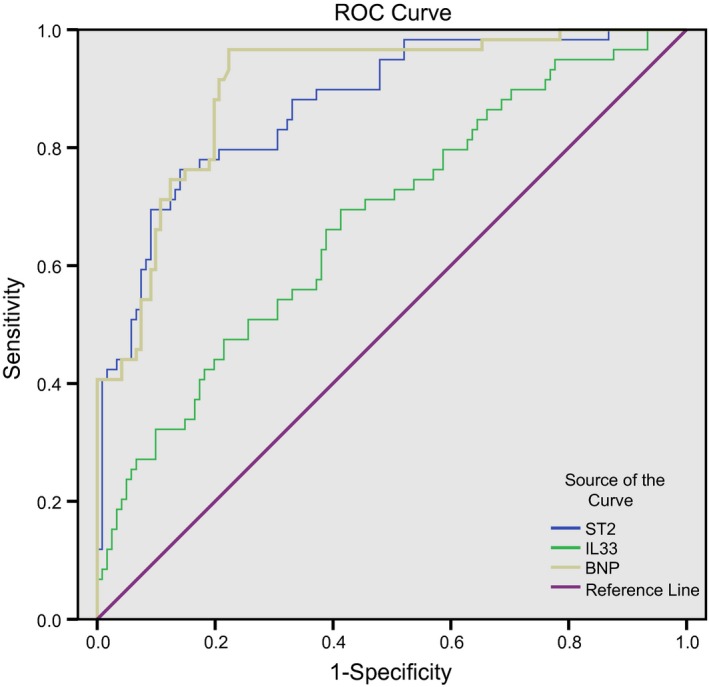
ROC curve analysis on predictive values of serum levels of ST2, IL‐33 and BNP for MACEs. AMI, acute myocardial infarction; ST2, homolog of sulfotransferase; IL‐33, interleukin‐33; BNP, B‐brain natriuretic peptide.

The area under the ROC curve of ST2 at admission in predicting MACEs was 0.872 (95% CI: 0.82–0.93, *P *<* *0.05). The cut‐off value was 733.82 pg/ml, the sensitivity was 76.27%, and specificity was 85.92%. The area under the ROC curve of IL‐33 at admission in predicting MACEs was 0.675 (95% CI: 0.59–0.76, *P *<* *0.05). The cut‐off value of IL‐33 was 387.75 pg/ml, the sensitivity was 69.49%, and specificity was 58.68%. The area under the ROC curve of BNP at admission in predicting MACEs was 0.902 (95% CI: 0.86–0.95, *P *<* *0.05). The cut‐off value of BNP was 285.73 pg/ml, the sensitivity was 96.61%, and specificity was 77.69% (Fig. 4, Table 2).

**Table 2:** Cut‐off values of serum levels of ST2, IL‐33 and BNP in predicting the occurrence of MACEs

	AUC	95% CI	Cut‐off values	Sensitivity (%)	Specificity (%)	Predictive positive rate (%)	Predictive negative rate (%)
ST2	0.872	0.82–0.93	733.82	76.27	85.95	72.58	88.14
IL‐33	0.675	0.59–0.76	387.75	69.49	58.68	45.05	79.78
BNP	0.902	0.86–0.95	285.73	96.61	77.69	67.86	97.92

AUC, area under the curve; CI, confidence interval; MACEs, major adverse cardiovascular events; AMI, acute myocardial infarction; ST2, homolog of sulfotransferase; IL‐33, interleukin‐33; BNP, B‐brain natriuretic peptide.

### Logistic regression analysis of independent risk factors for MACEs in AMI patients

Logistic regression analysis was used to analyse the independent risk factors for MACEs in AMI patients, with MACEs as the dependent variable, while Gensini score, hs‐CRP, serum levels of ST2, IL‐33 and BNP as independent variables. The results demonstrated that the serum levels of ST2, IL‐33 and BNP at admission were independent risk factors for MACEs (all *P *<* *0.05), but Gensini score and hs‐CRP were not independent risk factors for MACEs (Table 3).

**Table 3:** Logistic regression analysis of independent risk factors for MACEs in AMI patients

Variable	B	S.E.	Wald	Exp (B)	Sig.	95% CI
ST2	0.009	0.002	21.102	1.009	<0.001	1.005–1.013
IL‐33	0.006	0.003	3.933	1.006	0.047	1.000–1.012
BNP	0.022	0.004	26.457	1.022	<0.001	1.014–1.031
Gensini score	0.019	0.013	1.913	1.019	0.167	0.992–1.046
hs‐CRP	0.599	0.310	3.729	1.821	0.053	0.991–3.347

B, regression coefficient; S.E., standard error of regression; Sig, significance; CI, confidence interval; MACEs, major adverse cardiovascular events; AMI, acute myocardial infarction; ST2, homolog of sulfotransferase; IL‐33, interleukin‐33; BNP, B‐brain natriuretic peptide; hs‐CRP, high‐sensitive C‐reactive protein.

### Comparison of the 1‐year overall survival rate in AMI patients between the MACEs and MACEs‐free groups

**Figure 5:**
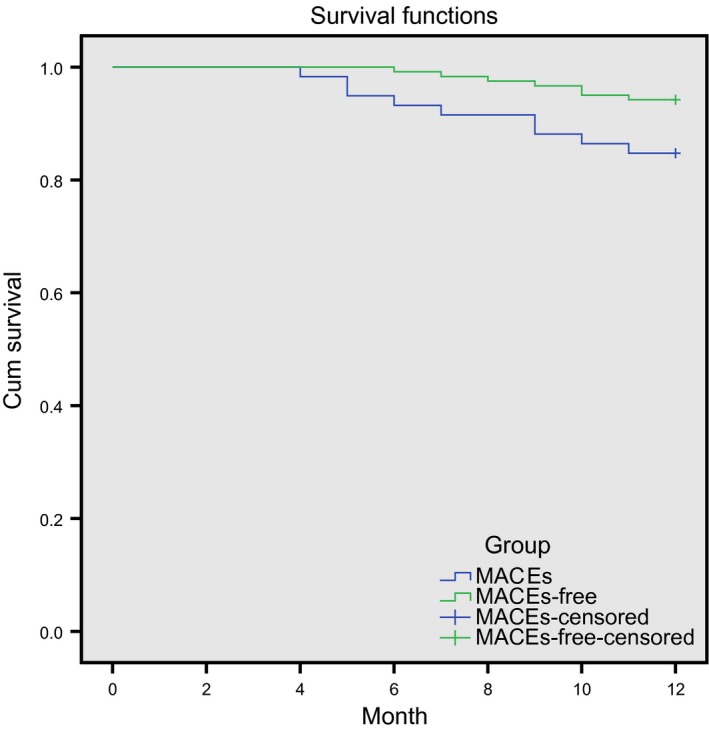
Comparison of the 1‐year overall survival rate in AMI patients between the MACEs and MACEs‐free groups. AMI, acute myocardial infarction; MACE, major adverse cardiovascular events.

The time‐point when PCI surgery was successfully performed was taken as the starting point, and the occurrence of MACEs was taken as the end‐point. The effect of MACEs on the 1‐year overall survival rate of AMI patients was analysed. Within 1 year, nine patients died in the MACEs group, with the mortality rate 15.25% (9/59), while seven patients died in the MACEs‐free group, with the mortality rate 5.79% (7/121). The 1‐year overall survival rate was lower in the MACEs group than the MACEs‐free group (*P *<* *0.05; Fig. 5).

### Correlations of serum levels of ST2, IL‐33 and BNP with 1‐year overall survival rate in AMI patients

**Figure 6:**
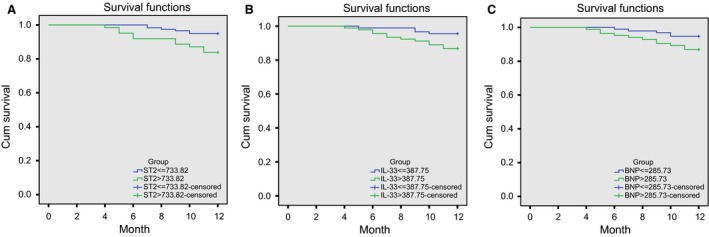
Kaplan–Meier curve analysis on the correlations of serum levels of ST2, IL‐33 and BNP with 1‐year overall survival rate in AMI patients. (A) correlations of serum level of ST2 with 1‐year overall survival rate in AMI patients; (B) correlations of serum level of IL‐33 with 1‐year overall survival rate in AMI patients; (C) correlations of serum level of BNP with 1‐year overall survival rate in AMI patients; AMI, acute myocardial infarction; ST2, homolog of sulfotransferase; IL‐33, interleukin‐33; BNP, B‐brain natriuretic peptide.

Taken the average serum levels of ST2, IL‐33, BNP of all the AMI patients as the dividing line, all patients were divided into low ST2 group (ST2 ≤ 733.82 pg/ml) and high ST2 group (ST2 > 733.82 pg/ml), low IL‐33 group (IL‐33 ≤ 387.75 pg/ml) and high IL‐33 group (IL‐33 > 387.75 pg/ml), and low BNP group (BNP ≤ 285.73 pg/ml) and high BNP group (BNP > 285.73 pg/ml). The 1‐year mortality rate was 5.08% (6/118) in the low ST2 group, 16.13% (10/62) in the high ST2 group, 4.49% (4/89) in the low IL‐33 group, 13.19% (12/91) in the high IL‐33 group, 4.17% (4/96) in the low BNP group and 14.29% (12/84) in the high BNP group. As Figure 6 suggested, the 1‐year overall survival rate was higher in AMI patients with low serum levels of ST2, IL‐33 and BNP than those with high serum levels of ST2, IL‐33 and BNP (all *P *<* *0.05).

## Discussion

As AMI is becoming a global acute disease which extremely threatens human health, initial biomarkers of AMI may be very important to improve the outcome. MACEs refer to complications occurring in AMI patients after operation, among which cardiogenic shock is related with a high mortality rate; therefore, a great deal of early treatments is under investigation to avoid or minimize MACEs 25, 26 . The study tried to explore the relationship of serum levels of ST2, IL‐33 and BNP with MACEs in patients with AMI undergoing PCI and found that serum levels of ST2, IL‐33 and BNP are potential in predicting MACEs in patients with AMI undergoing PCI.

First of all, the findings in our study indicate that serum levels of ST2, IL‐33 and BNP are significantly higher in AMI patients with MACEs than those without MACEs, and that AMI patients with high serum levels of ST2, IL‐33 and BNP have a lower 1‐year survival rate. ST2 is a member of IL‐1 receptor family, and IL‐33 is a cytokine belonging to IL‐1 family; IL‐33 is identified as the specific functional ligand of ST2 and the binding of IL‐33 to cells which express ST2 might result in nuclear factor kappa B (NF‐kB) and MAP kinases activation, leading to the induction of TH2 cytokines and balancing ongoing inflammatory TH1 responses 10. It is considered that sST2 is a decoy receptor of IL‐33, which inhibits the protective effects of the cytokine in diseases such as cardiac remodelling 27. That may be why the serum levels of IL‐33 and ST2 are significantly elevated in AMI patients with MACEs than those without MACEs. BNP or N terminal pro BNP (NT‐proBNP) is a polypeptide which is secreted by the ventricles of heart, in response to excessive stretching of cardiomyocytes or heart muscle cells, and increased BNP may be involved in a greater severity of myocardial ischaemic territory 16, 28, which may to some extent explain the positive correlation between elevated BNP and MACEs in AMI patients. Consistent with our study, another research about relationship of BNP and prognosis of coronary artery disease (CAD) also reveals that elevated serum BNP level is independently correlated with inducible ischaemia in patients diagnosed with stable CAD 29. Former studies also point out that ST2 and BNP are strikingly prognostic in heart failure disease 30, 31. Additionally, ST2 is reported to increase markedly in the early course of AMI, and the IL‐33/ST2 ratio is a reliable prognostic factor of AMI patients 1, indicating that high serum levels of ST2, IL‐33 and BNP might be related with low survival rate of AMI patients.

In addition, the results reveal that serum levels of ST2, IL‐33 and BNP are positively correlated with Gensini score. Gensini score, a quantitative digital method which shows the severity of CAD applied in combination with image analyses. It is reported that patients with impaired glucose tolerance showed higher Gensini score than those with normal glucose tolerance 32. BNP has been accepted as a valuable marker for the prediction of left ventricular dysfunction in the last decade, and AMI patients are demonstrated to have a significantly higher BNP level than patients with non‐cardiac chest pain or unstable angina 33. Shahabi *et al*. has pointed out that serum BNP level is a potent predictor for severity of CAD in the analysis of the predicting ability of NT‐proBNP based on Gensini scoring 28. Moreover, it has been proved in our previous statements that serum levels of ST2 and IL‐33 are high in AMI patients with MACEs, and a combined use of ST2 and NT‐proBNP can improve the predicting ability of heart failure death; when the serum levels of ST2 and IL‐33 are high, the AMI severity as well as Gensini score is elevated, in other words, the serum levels of ST2 and IL‐33 are positively correlated with Gensini score of AMI 27, 34.

In conclusion, serum levels of ST2, IL‐33 and BNP are high in AMI patients with MACEs, and AMI patients with high serum levels of ST2, IL‐33 and BNP have a low 1‐year survival rate. Besides, positive correlations are found in serum levels of ST2, IL‐33 and BNP and Gensini score. Therefore, we may conclude that serum levels of ST2, IL‐33 and BNP may have great predictive value in MACE after PCI in patients with AMI. However, due to the limitation of study time, only a 1‐year follow‐up in AMI patients was conducted in our study, further studies with more long‐term observations are still needed to determine the predictive value of serum levels of ST2, IL‐33 and BNP in AMI patients.

## Competing interests

The authors have declared that no competing interests exist.
